# Prevalence and correlates of depressive symptoms in adult patients with pulmonary tuberculosis in the Southwest Region of Cameroon

**DOI:** 10.1186/s40249-016-0145-6

**Published:** 2016-06-02

**Authors:** Jules Kehbila, Cyril Jabea Ekabe, Leopold Ndemnge Aminde, Jean Jacques N. Noubiap, Peter Nde Fon, Gottlieb Lobe Monekosso

**Affiliations:** Department of Medicine, Faculty of Health Sciences, University of Buea, Buea, Cameroon; Clinical Research Education, Networking and Consultancy, BP 3480 Douala, Cameroon; School of Public Health, Faculty of Medicine and Biomedical Sciences, University of Queensland, Brisbane, Australia; Department of Medicine, Groote Schuur Hospital and University of Cape Town, Cape Town, South Africa; Medical Diagnostic Center, Yaoundé, Cameroon; Department of Public Health and Hygiene, Faculty of Health Sciences, University of Buea, Buea, Cameroon; Department of Medicine, Faculty of Health Sciences, University of Buea and Global Health Dialogue Foundation, Buea, Cameroon

**Keywords:** Prevalence, Correlates, Depression, Pulmonary tuberculosis, Cameroon

## Abstract

**Background:**

Tuberculosis (TB) remains a global health challenge and depression is a significant contributor to the global burden of disease. Current evidence suggests that there is an association between depressive symptoms and TB, lower adherence to treatment, and increased morbidity and mortality. However, there is paucity of data regarding these associations in Cameroon. This study aimed to determine the prevalence and correlates of depression in adult patients with pulmonary TB (PTB) in the Southwest Region of Cameroon.

**Methods:**

A hospital-based cross-sectional study involving 265 patients with PTB was conducted from 2^nd^ January to 31^st^ March 2015 in the Limbe Regional Hospital and the Kumba District Hospital. Depression was diagnosed using the standard nine-item Patient Health Questionnaire, and classified as none, mild or moderate. Logistic regressions were used to investigate correlates of depression in these patients.

**Results:**

Of the 265 patients (mean age 36.9 ± 10 years) studied, 136 (51.3 %) were female. The prevalence of depression was 61.1 % (95 % *CI*: 55.1–66.8), with a significant proportion (36.6 %) having mild depression. Multivariable logistic regression analysis showed that being female (a*OR* = 3.0, 95 % *CI* (1.7–5.5), *P* < 0.001), having a family history of mental illness (a*OR* = 2.5, 95 % *CI*: 1.3–5.4, *P* > 0.05), being on retreatment for TB (a*OR* = 11.2, 95 % *CI*: 5.2–31.1, *P* < 0.001), having discontinued treatment (a*OR* = 8.2, 95 % *CI*: 1.1–23.3, *P* < 0.05) and having a HIV/TB co-infection (a*OR* = 2.5, 95 % *CI*: 1.2–6.5, *P* < 0.001) were factors associated with having a higher chance of being depressed.

**Conclusion:**

Our study suggests that there is a high prevalence of depression among PTB patients, with more than one in two patients affected. Multidisciplinary care for TB patients involving mental health practitioners is highly encouraged, especially for high-risk groups.

**Electronic supplementary material:**

The online version of this article (doi:10.1186/s40249-016-0145-6) contains supplementary material, which is available to authorized users.

## Multilingual abstracts

Please see Additional file 1 for translations of the abstract into six official working languages of the United Nations.

## Background

Tuberculosis (TB) remains a leading infectious cause of morbidity and mortality worldwide. The recent 2013 global incidence of TB is nine million, with more than half (56 %) of those infected residing in Southeast Asia and the Western Pacific regions. A further quarter is in the African region, which also had high prevalence and mortality rates [1]. There has been a slow but steady decrease in the incidence of TB in Cameroon from 260 per 100 000 people in 2010 to 235 per 100 000 as of 2014 [2].

Anxiety disorders and depression are the most prevalent mental disorders in the general population. Depression is a common mental disorder characterised by loss of interest or pleasure, feelings of guilt or low self-worth, disturbed sleep or appetite, low energy and poor concentration, insomnia or hypersomnia and occasionally suicidal thoughts [3, 4]. Depression affects 350 million people globally and a health survey conducted by the World Health Organization (WHO) in 2012, in 17 countries revealed that one in 20 people reported depression [3]. According to a WHO report on global burden of disease (GBD) in 2008, depression ranks fourth and is predicted that, by 2030 it might be the leading burden of disease worldwide [5]. Depression can lead to suicide, which accounts for the loss of one million lives per year and 30,000 deaths/day globally [3, 6].

The prevalence of depression in TB patients is reported to be very high, reaching as high as 95.5 % in Nigeria [7]. Data on the magnitude of depression in TB patients in Cameroon are scarce, however, a study conducted in Yaoundé by Balkissou et al. reported a 30.9 % prevalence of depression in TB patients [8]. Furthermore, another study in Yaoundé showed a high prevalence of depressive symptoms (63 %) in patients infected with HIV, a disease commonly associated with TB [9].

Depression has also been identified as a co-morbid condition in TB and is associated with antibiotic drug resistance, high rates of community transmission [9, 10], and an increased level of morbidity and mortality [10, 11]. Depressed individuals with TB are less likely to seek care promptly and those who do are significantly less likely to take medications consistently and/or completely [10–12]. This can lead to drug resistance, morbidity and mortality [13–16]. Therefore, depression may be an unidentified driver of TB and multidrug resistant TB epidemics. Understanding the relationship between depression, TB and disease outcomes is crucial for developing efficient strategies to tackle the persistent burden of TB, especially in endemic communities. This study was conducted to determine the prevalence and factors associated with depression in patients with pulmonary tuberculosis (PTB) in the Southwest Region of Cameroon.

## Methods

### Study design and sites

This was a hospital-based cross-sectional study, conducted from 2^nd^ January to 31^st^ March 2015 with PTB patients in the Limbe Regional Hospital and the Kumba District Hospital, two referral hospitals for TB treatment in the Southwest Region of Cameroon. These hospitals are well equipped for the diagnosis, management and follow-up of patients with TB, and offer Directly Observed Treatment Short course (DOTS) services.

### Study participants

The study participants were selected from all patients diagnosed with PTB according to the National Tuberculosis Control Programme guidelines in Cameroon. Newly diagnosed PTB patients or those undergoing retreatment for at least two weeks were included in the study. Patients with extra-PTB, those aged < 21 years, or who were pregnant, critically ill or presenting severe complications such as respiratory distress or a mental illness (like Alzheimer’s disease) that could impair memory were excluded.

### Sample size and sampling

A convenient sampling method was employed. All patients who met the inclusion criteria and were receiving care at the study sites who consented to participate in the study were considered. Out of the 280 patients who received care in these hospitals during the study period, 265 were included in the study.

### Data collection

Data were collected by two physicians (JK and CJE), who were assisted by nurses working in the TB units of the respective study hospitals. Patients with PTB were interviewed using a structured questionnaire containing questions on the socio-demographic and disease characteristics of the participants. Socio-demographic variables included: age, sex, marital status, educational level, place of residence, employment status and occupation. Disease specific characteristics included: sputum type, patient status (hospitalised or outpatient), method of treatment, presence of an associated co-morbidity and HIV status.

Depression was assessed using the nine-item Patient Health Questionnaire (PHQ-9), which was administered in English. The PHQ-9 is a validated tool for the assessment of depression in clinical practice, based on the criteria outlined in the Diagnostic and Statistical Manual of Mental Disorders, 4th edition [17, 18]. It has good psychometric properties compared to other validated instruments and has been widely used in clinical practice and research. It has a sensitivity and specificity of 88 % for major depression [19], and a cut-off score on a scale of 10 is diagnostic of major depression [20]. It requires participants to rate, how often and for how long they have been bothered by any of the listed problems during the previous two weeks. The score for each question varies from 0 to 3 (0 = not at all, 1 = several days, 2 = more than half of the days, 3 = nearly every day), with a result range of 0–27. A score of 0–4 indicates ‘no depression’, 5–9 indicates ‘mild depression’, 10–14 indicates ‘moderate depression’, 15–19 indicates ‘moderately severe depression’ and 20–27 indicates ‘severe depression’. This was re-categorised as follows: no depression (score 0–4), mild depression (score 5–14) and moderate depression (score 15–27). This was adapted from studies by Basu et al. [21], Issa et al. [22] and Masumoto et al. [23].

### Data management and analysis

The data collected were cross checked at the end of every week, entered into and analysed using IBM SPSS statistical software v.16 for Windows (SPSS Inc., Chicago, IL, USA). The prevalence of depression in PTB patients was calculated as a proportion of PTB patients with a score greater than 4 (on the PHQ-9) out of the total number of PTB patients.

We summarised continuous variables using means and standard deviations (SDs), and categorical variables using frequencies and percentages. The chi-square test was conducted to determine the associations between potential predictor variables and depression. A multivariable logistic regression analysis was conducted to investigate factors independently associated with depression. A *P*-value < 0.05 was considered statistically significant.

### Ethical consideration

Ethical clearance was obtained from the Faculty of Health Sciences Institutional Review Board of the University of Buea, Cameroon. The administrators of the study hospitals also authorised the study. Written informed consent was obtained from all the participants. Patient confidentiality was maintained and the study adhered to the Declaration of Helsinki. Patients with moderate to severe depression were referred to a psychiatrist for further evaluation and management.

## Results

### General characteristics of the study population

#### Socio-demographic characteristics

From a pool of 280 potential participants, 15 were excluded: five due to a language barrier (natives who could not understand English), seven due to respiratory distress and three due to hoarseness of voice. The response rate was thus 94.6 %.

Among the 265 patients retained, 136 (51.3 %) were female. Ages ranged from 21 to 66 years, with mean of 37.0 years (*SD*: 10.1). The modal age group was 31–40, representing 38.5 % of the study population. Over half (52.5 %, *n* = 139) of the study population was single. The majority (45.7 %) of the patients had attended secondary school and 61.5 % of the participants were urban dwellers. Table 1 summarises the socio-demographic characteristics of the study population.

**Table 1:** Socio-demographic characteristics of adult PTB patients at the Limbe Regional and Kumba District Hospitals, Southwest Region of Cameroon, 2015

Variable	Description	*N* (%)
Age	21–30	67 (25.3)
	31–40	102 (38.5)
	41–50	62 (23.4)
	>51	34 (12.8)
Sex	Male	129 (48.7)
	Female	136 (51.3)
Marital status	Married	98 (37)
	Single	139 (52)
	Co-habiting	24 (9.1)
	Widow/widower	2 (0.8)
	Divorced/separated	2 (0.8)
Occupation	Farmer	76 (28.7)
	Trader	59 (22.3)
	Student	16 (6)
	Housewife	24 (9.1)
	Artist	6 (2.3)
	Teacher	38 (14.3)
	Other	46 (17.4)
Educational level	No school	18 (6.8)
	Primary school	112 (42.3)
	Secondary school	121 (45.7)
	Tertiary	14 (5.3)
Place of residence	Urban	162 (61.1)
	Rural	102 (38.5)
Living alone	Yes	60 (22.6)
	No	205 (77.5)

#### Clinical characteristics

A greater proportion (60 %, *n* = 159) of patients were outpatients and the majority (77 %, *n* = 204) were sputum positive TB cases. A large proportion of the participants (63.8 %, *n* = 169) were of normal weight (18.5 < body mass index (BMI) < 25.5Kg/m^2^). Over half of the patients (55.1 %, *n* = 146) were in the intensive phase (the first two months) of their treatment. About four-fifths (80.4 %) were new cases, while the rest were retreatment cases. Forty-six (17 %) participants reported to have stopped their treatment at some point; six (2 %) had stopped less than a month ago, 20 (7.5 %) stopped between one and two months ago and 20 (7.5 %) had completely stopped.

With respect to their initial clinical presentations, nearly half of the patients (*n* = 124) exhibited less than four symptoms. The most common symptom was cough (57.0 %), followed by fatigue (15.4 %), shortness of breath (10.6 %), fever (9.9 %), weight loss (3 %) and others (4 %). One hundred participants (37.7 %) had a co-morbidity, among which 88 (88 %) had HIV/AIDS, three (3 %) had heart-related diseases, three (3 %) had asthma and five (5 %) had others, and lastly a case of chronic obstructive pulmonary disease. Only a quarter of the patients (25.6 %) reported a family history of mental disease. Table 2 summarises some of the clinical characteristics of the study population.

**Table 2:** Clinical characteristics of patients with PTB at the Limbe Regional and Kumba District Hospitals, Southwest Region of Cameroon, 2015

Variable	Description	*N* (%)
Sputum smear at time of diagnosis	Positive	204 (77)
	Negative	61 (23)
Treatment status	New case	213 (80.4)
	Retreatment c	52 (19.6)
Treatment phase	Intensive	146 (55.1)
	Continuation	119 (44.9)
Patient status at time of study	Hospitalised	106 (40)
	Outpatient	159 (60)
Other co-morbid conditions	Yes	100 (37.7)
	No	165 (62.3)
TB/HIV co-infection	Yes	88 (33.2)
	No	177 (66.8)
Family history of mental illness	Yes	68 (25.7)
	No	197 (64.3)

### Prevalence of depression

The overall prevalence of depressive symptoms in TB patients was 61.1 % (95 % *CI* = 55.1–66.8); 103 (38.9 %) patients had no depression, 97 (36.6 %) had mild depression and 65 (24.5 %) had moderate depression. Females were more affected than males (38.5 % vs. 22.6 %). Major depression was found in 41.5 % (*n* = 110) of the patients, with 27.9 % (*n* = 74) of those female and 13.6 % (*n* = 36) male.

### Factors associated with depression

#### Univariate analysis

A cross tabulation using a chi-square test (see Tables 3 and 4) showed a significant relationship between depression and the following variables: sex (*P* < 0.001), BMI (*P* < 0.001), sputum smear type (*P* < 0.05), treatment phase (*P* < 0.05), treatment status (*P* < 0.05), stopping treatment before completion (*P* = 0.001) and time since stopping (*P* = 0.001), patient status at time of study (*P* = 0.001), those with other co-morbidities *(P* < 0.001), with HIV/AIDS being the most common (87.9 %), and family history of mental illness (*P* < 0.001).

**Table 3:** Association of depression and its severity with socio-demographic variables of patients with PTB in the Southwest Region of Cameroon, 2015

Variable	Severity of depression, *N* (%)			*p*-value
	None	Mild	Moderate	
Sex				
Male	69 (53.5)	39 (30.2)	21 (16.3)	<0.001
Female	34 (25.0)	58 (42.8)	44 (32.4)	
Age				
21–30	29 (10.9)	20 (7.5 %)	18 (6.8)	<0.001
31–40	38 (14.3)	44 (16.6)	20 (7.5)	
41–50	22 (8.3)	14 (5.3)	26 (9.8)	
> 51	14 (5.3)	19 (7.2)	1 (0.4)	
Living alone				
Yes	15 (5.7)	16 (6)	29 (10.9)	0.16
No	88 (33.2)	81 (30.6)	36 (13.6)

**Table 4:** Association between clinical characteristics and depression and its severity in patients with PTB in the Southwest Region of Cameroon, 2015

Variable	Severity of depression, *N* (%)			*P*-value
	No depression	Mild	Moderate	
BMI				
Underweight	22 (8.3)	22 (8.3)	34 (12.8)	<0.001
Normal weight	73 (27.5)	65 (24.5)	31 (47.7)	
Overweight	8 (3)	10 (3.8)	0	
Sputum smear				
Positive	77 (29.1)	69 (26)	58 (21.9)	0.022
Negative	69 (26.0)	28 (10.6)	7 (2.6)	
Treatment status				
New case	95 (35.8)	78 (29.4)	40 (15.1)	0.016
Retreatment	78 (29.4)	19 (7.2)	25 (9.4)	
Stopped treatment before completion				
Yes	9 (3.4)	17 (6.4)	20 (7.5)	0.001
No	94 (35.5)	80 (30.2)	45 (17.0)	
Patient status				
Hospitalised	35 (13.2)	32 (12.1)	39 (14.7)	0.001
Outpatient	68 (25.7)	65 (24.5)	26 (9.8)	
Severity of symptoms				
No symptoms	23 (8.7)	16 (6.0)	0	0.073
< 4 symptoms	51 (19.2)	55 (20.8)	28 (10.6)	
> 4 symptoms	29 (10.9)	26 (9.8)	37 (14)	
Co-morbidities				
Yes	21 (7.9)	39 (14.7)	40 (15.1)	<0.001
No	82 (30.9)	58 (21.9)	25 (9.4)	
Family history of mental illness				
Yes	15 (5.7)	24 (9.1)	29 (10.9)	<0.001
No	88 (33.2)	29 (10.9)	36 (13.6)	

#### Multivariate analysis

The multivariable logistic regression analysis showed that out of the 10 factors investigated (see Table 5), the following were found to be independently associated with depression (these patients were more likely to be depressed): being female (adjusted odd ratio, a*OR* = 3.0 [1.7–5.5], *P* < 0.001); on retreatment for PTB (a*OR* = 11.2 [5.2–31.1], *P* < 0.001); a family history of mental illness (a*OR* = 2.5 [1.3–5.4], *P* < 0.05); discontinuing treatment (a*OR* = 8.2 [1.1–23.3], *P* = 0.05) and a HIV/TB co-infection (a*OR* = 2.5 [1.3–6.5], *P* < 0.01).

**Table 5:** Multivariable logistic regression analysis showing factors independently associated with depression in PTB patients in the Southwest Region of Cameroon, 2015

Variable	Multivariate analysis	
	*OR* (95 % *CI*)	*P*-value
Age		
> 40 years	1.3 (0.7–2.4)	0.417
≤ 40 years	Ref	
Sex		
Female	3.0 (1.7–5.5)	<0.001
Male	Ref	
Co-morbidity		
Yes	2.5 (1.2–6.5)	<0.001
No	Ref	
BMI		
< 18.5 kg/m2	0.3 (0.1–1.3)	0.105
18.5–24.9 kg/m	0.4 (0.1–1.4)	0.122
≥ 25 kg/m2	Ref	
Treatment phase		
Intensive	1.2 (0.6–2.1)	0.723
Continuation	Ref	
Family history of mental illness		
Yes	2.5 (1.3–5.4)	0.013
No	Ref	
Patient status		
Hospitalised	1.2 (0.6–2.3)	0.677
Outpatient	Ref	
Sputum smear		
Positive	1.9 (0.9–3.8)	0.121
Negative	Ref	
Discontinuation of treatment		
Yes	8.2 (1.1–23.3)	0.048
No	Ref	
Treatment status		
Retreatment	11.2 (5.2–31.1)	<0.001
New	Ref	

## Discussion

Depression is a significant contributor to the GBD and a major problem for TB patients, leading to non-adherence of treatment and drug resistance [24]. The long-term goal of the 2012 World Mental Health Day was to establish parity for mental health with physical health in national health priorities and services [3]. Yet there is still paucity of data on mental health in Cameroon, with this study being one of the few existing on depression in TB patients, and the first such study conducted in the Southwest Region of the country.

### Prevalence of depression

The prevalence of depression in patients with PTB in our study was high, with over half (61.1 %) of the participants affected. This is similar to findings from India, Iran and Ethiopia, with prevalence rates ranging between 54.2 and 67.1 % [25–27]. Even higher rates have been reported in Pakistan (72 %), South Africa (81 %) and India (82 %) [28–30]. However, slightly lower prevalence rates ranging from 19.2 to 45.5 % have been observed in Ethiopia, Cameroon, India, Peru and Nigeria [7, 8, 22, 25, 31, 32].

These findings suggest the alarming burden of depression in TB patients. The high prevalence in our study may in part be due to the fact that we investigated depressive symptoms rather than major depressions, as the other studies have. Secondly, this could also be accounted for by the high sensitivity and specificity of the screening tool (PHQ-9) used in our study. Differences in study settings and patient characteristics could also partly account for the variation in prevalence rates for depression across studies. It should be noted, however, that over a third of TB patients are likely to suffer from a depressive illness and hence the mental health of these patients should not be overlooked. Another reason why this study showed a high prevalence rate may be the fact that there was a strong association between depression and method of treatment, with 67 % of the hospitalised patients being depressed and the majority having severe infections. The stigma of isolation, association between depression and disease severity, coupled with a presence of co-morbidities (particularly HIV/TB co-infection) were probably the drivers of the high prevalence in this group of patients [33].

### Factors associated with depression

An association between depression and sex was observed in our study, with 75 % of females being depressed. This is similar to studies conducted by Balkissou et al. in Yaoundé, Cameroon, Masumoto et al. in the Philippines, Aamir and Aisha in Pakistan, and Panchal and co-workers in India [8, 23, 29, 30]. The predisposition of females could possibly be due to the association between female hormonal factors and depression [4, 10, 34].

In our study, BMI was a significant factor associated with depression in PTB patients, with it showing an inverse relationship with depression in the bivariate analysis. Patients with a BMI <18.5 kg/m^2^ had the highest level of moderate depression (*P* < 0.001), which is similar to a study conducted by Masumoto et al. in Manila, Philippines (*OR* 2.16, 95 % *CI*: 1.25–3.73, *P* < 0.01) [23]. However, BMI was not a predictive factor of depression in PTB patients in the multivariable analysis, and hence the influence of BMI on depression is yet to be investigated.

Overall, 100 (37.7 %) of the studied TB patients had an associated chronic illness (87.9 % with HIV/AIDS) and depression was more frequent (79 %) in patients with co-morbidities. In fact, patients with a HIV/TB co-infection where 2.5 times more likely to be depressed than those without (a*OR* 95%CI = 2.5 [1.2–6.5], *P* < 0.001) a co-infection. This is similar to studies done by Adem et al. in Ethiopia [25], Davoodian et al. in Iran and Peltzer et al. in South Africa, but unlike in our study, the latter two found a poor predictive ability in the multivariate analysis [27, 28]. This is probably due to the strong association between HIV and depression, which has been reported by other studies in our setting [9].

Patients with PTB who have a family history of a mental illness were more likely to be depressed. In our study, 68 (26 %) participants had a positive family history of mental disorder, with about 77.9 % of them being depressed. The multivariate analysis showed that patients with a family history of mental illness were 2.5 times more likely to be depressed as compared to their counterparts. Similar results were obtained in a study conducted in Iran by Davoodian et al. [27]. This finding could be attributed to the association between genetics and depression [4, 34, 35], but few studies have attempted to investigate this relationship with almost none conducted in our setting.

The prevalence of depression was higher in patients in the age group of 31–40 years though this association was not significant in the multivariate analysis. This is similar to the findings of Masumoto et al. in Manila with the age group of 31–50 years most affected [23], though unlike the higher age groups reported in other studies by Ige et al. in Nigeria, Basu et al. in India, Adem et al. in Ethiopia and Ugarte-Gil et al. in Peru [7, 21, 25, 32]. The finding in our study is most probably due to the fact that the majority of participants were youths with a high prevalence of HIV/TB co-infection. Secondly, our study was conducted in a predominantly urban setting where the active and working age group is dominated by youths due to urbanisation.

Slightly more than half of the study participants were single (52.5 %), with 59 % of them being depressed. This was similar to the findings of Basu et al. in India [21]. Unlike our study, other studies have shown a higher prevalence of depression in partners co-habiting [7], as well as in divorced couples [29, 30, 36].

No significant relationship was observed between the level of education and depression in our study (*P* > 0.05), even though the prevalence of depression was higher in those who had never attended school. This is similar to studies conducted in India by Purohit et al. and in Pakistan by Aamir and Aisha [26, 29], and could be accounted in part by the high association between illness perception of TB and level of depression [33]. Patients being treated for PTB who had discontinued their treatment at one point and were being retreated, or continued their treatment had significantly higher odds of being depressed. Depression may have been the reason why these patients stopped their treatment in the first place and the difficulties associated with retreatment (type of drug and duration of treatment) could make adherence difficult.

### Study limitations

This study was cross-sectional by design and could not establish actual risk factors of depression in TB patients. There is also the possibility of overestimation of the prevalence of depression due to the fact that the PHQ-9 contains some symptoms of TB.

We also acknowledge the fact that alcohol and substance abuse, which are potential factors that have been associated with depression elsewhere, was not considered in our study. This is because they are not common correlates in our setting [9].

Our study is, however, one of the pioneer studies in Cameroon on depression and TB. The tool used to diagnose depression in our study, the PHQ-9, is a standard screening tool for depressive symptoms, which has previously been validated in other settings similar to ours [37–39], for the diagnosis of depression. Moreover, the PHQ-9 has high sensitivity and specificity, thus enhancing confidence and comparability of our results.

## Conclusion

Overall, our findings indicate a high prevalence of depressive symptoms in PTB patients in the Southwest Region of Cameroon, especially among females, those with a family history of a mental disorder, those on retreatment for TB, those who discontinued treatment and those who have a co-infection with HIV (independent correlates). While multidisciplinary care is encouraged for this vulnerable group, larger multicentre cohort studies are warranted to explore and fine-tune the observations from our study. Our study not only highlights the need for mental services in TB units, but also for multidisciplinary and holistic care for TB patients. Interventions for screening and control of mental health should be intensified, especially in this vulnerable population. We recommend that further studies are conducted on depression and drug resistance.

## Additional file

Additional file 1: Multilingual abstracts in the six official working languages of the United Nations. (PDF 279 kb)

## Abbreviations

a*OR*: adjusted odd ratio
BMI: body mass index
GBD: global burden of disease
PHQ-9: nine-item Patient Health Questionnaire
PTB: pulmonary tuberculosis
SD: standard deviation
TB: tuberculosis
WHO: World Health Organization
