# Supplementary material for: Prevalence and correlates of depressive symptoms in adult patients with pulmonary tuberculosis in the Southwest Region of Cameroon
Source: Infect Dis Poverty. 2016 Jun 2;5:51. doi: 10.1186/s40249-016-0145-6 (PMC4895984; doi:10.1186/s40249-016-0145-6)
Supplement: Additional file 1: — Multilingual abstracts in the six official working languages of the United Nations. (PDF 279 kb) [file 40249_2016_145_MOESM1_ESM.pdf]

Translation of the abstract into the five official working languages of the United Nations

انتشار العدوى وارتباط أعراض الاكتئاب بمرض السل الرئوي عند المرضى البالغين في منطقة الجنوب الغربي للكاميرون

جولس كابيلا، سيريل جابيا ايكابي، ليوبولد اند منج أميند، جان جاك ن نوبياب، بيتر اند فون، غوتليب لوب موناكوسو

ملخّص

تقديم عام: لا يزال مرض السل يُشكل تحديا عالميا على مستوى الصحّة. تعتبر حالة الاكتئاب من أهم العوامل المتسببة في عبء المرض العالمي، وقد أثبتت الأدلة الحالية وجود علاقة بين أعراض الاكتئاب ومرض السل وضعف الالتزام بالعلاج وتزايد نسبة الحالات المرضية والوفيات. إلا أنّه توجد ندرة في البيانات المتعلقة بهذه العوامل في الكاميرون إذ تهدف هذه الدراسة إلى تحديد مدى انتشار هذا المرض وارتباطه بالاكتئاب عند المرضى البالغين المصابين بالسل الرئوي في منطقة جنوب غرب الكاميرون.

التقنيات المستخدمة:تم إجراء الدراسة اعتمادا على طريقة الدراسات المقطعية على 265 مصاب بالسل الرئوي من 2 يناير إلى 30 مارس 2015 بالمتشفى الإقليمي في ليمب ومستشفى كيمبا. تم تشخيص الاكتئاب اعتمادا على معيار البند التاسع من الاستبيان المتعلق بصحة المريض ويتمّ تصنيف حالة المريض كسليم أو في حالة اكتئاب خفيفة أو حالة اكتئاب معتدلة. وتمّ استخدام الانحدار اللوجستي (logistic regressions) للكشف عن العوامل المرتبطة بحالات الاكتئاب عند هؤلاء المرضى.

النتائج: من بين 265 مريض أُجريت عليهم الدراسة (متوسط العمر 36.9±10 سنوات) كان من ضمنهم 136 (51.3%) مريضا من الإناث. بلغت نسبة انتشار حالة الاكتئاب 61.1% (95% *CI*: 55.1–66.8) مع نسبة هامة (36.6%) ممن يعانون من اكتئاب خفيف. أثبتت تحاليل الانحدار اللوجستي متعدد المتغيرات (Multivariable logistic regression) أن تكون أنثى (a*OR*=3.0,95% *CI* (1.7-5.5), *P*<0.001) أو في عائلة عانت من الأمراض العقلية في الماضي (a*OR*=2.5, 95% *CI*: 1.3-5.4, *P*>0.05) أو أن تكون في مرحلة إعادة معالجة مرض السل(a*OR*=11.2, 95% *CI*: 5.2-31.1, *P*<0.001) أو تمرّ بمرحلة علاج على مراحل متفرقة (a*OR* = 8.2, 95% *CI*: 1.1–23.3, *P*<0.05) وتعاني من فيروس نقص المناعة البشرية إضافة إلى مرض السل (a*OR*=2.5,95% *CI*:1.2-6.5, *P*<0.001) فإنّ كلّ هذه العوامل تُعتبر سببا مساعدا في ارتفاع احتمال الإصابة بحالة الاكتئاب.

الخاتمة: تشير دراستنا إلى وجود نسبة مرتفعة لانتشار حالة الاكتئاب بين صفوف المرضى المصابين بمرض السل الرئوي بمعدّل إصابة شخص من ضمن شخصين. يتم التشجيع على اعتماد الرعاية متعددة التخصصات لمرضى السل وخاصة للفئات الأكثر عرضة للخطر بإشراك الأطباء المتخصصين في مجال الصحة النفسية.

Translated from English version into Arabic by Zeineb TRABELSI, through

TRANSLATORS WITHOUT BORDERS

喀麦隆西南部成年肺结核患者抑郁的发生情况及相关因素

Jules Kehbila, Cyril Jabea Ekabe, Leopold Ndemnge Aminde, Jean Jacques N Noubiap, Peter Nde Fon, Gottlieb Lobe Monekosso

摘要

引言：结核病仍然是全球卫生的挑战，而抑郁也是全球疾病负担的一个重要来源。有证据表明抑郁和结核病的服药依从性降低、发病率和死亡率增加有关联。但是，喀麦隆缺乏这方面相关性的数据。本研究旨在弄清喀麦隆西南部成年肺结核患者的抑郁发生情况及相关因素。

方法：2015 年 1 月 2 日-3 月 31 日期间开展了一项基于医院的横断面调查，纳入了 Limbe 和 Kumba 两家区级医院的 265 例肺结核病例。采用病人健康问卷抑郁量表对抑郁进行诊断，并分为无、轻度和中度 3 级。应用 Logistic 回归方法分析以上患者的抑郁相关因素。

结果：265 例研究对象平均年龄 36.9 岁（标准差 10 岁），其中女性 136 例（51.3%）。抑郁发生率为 61.1%（95% *CI*：55.1-66.8），其中轻度占比 36.6%。多变量 Logistic 回归分析表明，以下变量是抑郁发生的危险因素，分别为女性（调整 *OR*=3.0，95% *CI*：1.7–5.5，$P<0.001$），家族精神病史（调整 *OR*=2.5，95% *CI*：1.3–5.4，$P>0.05$），正在接受抗结核治疗（调整 *OR*=11.2，95% *CI*：5.2–31.1，$P<0.001$)，抗结核治疗曾中断过（调整 *OR*=8.2，95% *CI*：1.1–23.3，$P<0.05$），同时感染 HIV 和结核（调整 *OR*=2.5，95% *CI*：1.2–6.5，$P<0.001$）。
结论：本研究表明肺结核患者抑郁发生率较高，占比超过半数。应积极鼓励对那些高危人群进行包括精神科医生在内的多学科照料。

Translated from English version into Chinese by Yang pin, through

CHINESE CENTER FOR DISEASE CONTROL AND PREVENTION
NATIONAL INSTITUTE OF PARASITIC DISEASES

Prévalence et corrélats des symptômes dépressifs chez les patients adultes atteints de tuberculose pulmonaire dans la région Sud-Ouest du Cameroun

Jules Kehbila, Cyril Jabea Ekabe, Leopold Ndemnge Aminde, Jean Jacques N Noubiap, Peter Nde Fon, Gottlieb Lobe Monekosso

Résumé

Contexte: La tuberculose reste un défi mondial de santé publique et la dépression joue un rôle significatif dans le fardeau mondial de la maladie. Des données récentes suggèrent une association entre les symptômes dépressifs et une moins bonne observance du traitement ainsi qu'une morbidité et une mortalité accrues dans la tuberculose. Les données relatives à ces associations sont cependant peu abondantes au Cameroun. La présente étude visait à déterminer la prévalence et les corrélats des symptômes dépressifs chez des patients adultes atteints de tuberculose pulmonaire dans la région Sud-Ouest du Cameroun.
Méthodes: Une étude transversale à l'hôpital portant sur 265 patients atteints de tuberculose pulmonaire a été menée du 2 janvier au 31 mars 2015 à l'Hôpital régional de Limbé et à l'Hôpital de district de Kumba. La dépression a été diagnostiquée à l'aide du Questionnaire sur la santé des patients à neuf rubriques et classée comme absente, légère ou modérée. Nous avons utilisé des régressions logistiques pour explorer les corrélats de la dépression chez ces patients.
Résultats: Sur les 265 patients étudiés (âge moyen 36,9±10 ans), 136 (51,3%) étaient des femmes. La prévalence de la dépression était de 61,1% (*IC* à 95%: de 55,1 à 66,8), avec une proportion significative (36,6%) de dépressions légères. L'analyse de régression logistique multivariée fait apparaître, comme facteurs de risque accru de dépression: le sexe féminin (*OR*a 3,0, *IC* à 95 % de 1,7 à 5,5), $P<0{,}001$), les antécédents familiaux de maladie mentale (*OR*a=2,5, *IC* à 95% de de 1,3 à 5,4, $P>0{,}05$), le fait d'avoir repris le traitement antituberculeux (*OR*a=11,2, IC à 95 % de 5,2 à 31,1, $P<0{,}001$), l'abandon du traitement (*OR*a=8,2, *IC* à 95 % de 1,1 à 23,3, $P<0{,}05$) et la co-infection VIH/tuberculose (*OR*a=2,5, *IC* à 95 % de 1,2 à 6,5, $P<0{,}001$).
Conclusion: Notre étude suggère une forte prévalence de la dépression parmi les patients souffrant de tuberculose pulmonaire: plus d'un patient sur deux est concerné. La prise en charge multidisciplinaire des patients tuberculeux, avec la participation de professionnels de la santé mentale, est fortement encouragée, en particulier pour les groupes à haut risque.

Translated from English version into French by Suzanne Assenat, through

Распространенность и корреляты депрессивных симптомов у взрослых больных туберкулезом легких в юго-западном регионе Камеруна

Жюль Кехбила, Кирилл Дабеа Экабе, Леопольд Ндемнге Аминде, Жан-Жак Н Ноубиап, Питер Нде Фон, Готтиеб Лобе Монекоссо

Аннотация

Цель: Туберкулез (ТБ) остается глобальной проблемой здравоохранения, и депрессия является существенным фактором глобального бремени болезней. Текущие данные свидетельствуют о том, что существует связь между депрессивными симптомами и туберкулезом, снижение приверженности к лечению, а также повышению уровня заболеваемости и смертности. Тем не менее, существует недостаток данных, касающихся этих связей в Камеруне. Целью данного исследования являлось определение распространенности и сопоставлять депрессии у взрослых пациентов с легочным туберкулезом (ЛТБ) в юго-западной области Камеруна.

Метод: Стационарное кросс-секционное исследование с участием 265 пациентов с ЛТБ был проведен со 2-го января по 31 марта 2015 года в областной больнице Лимбе и районной больницы Кумба. Депрессию был поставлен диагноз с применением стандартного опросника здоровья пациента состоящий из девяти пунктов, и классифицируется как отрицательной, легкой или умеренной. Логистическая регрессия была использована для изучения коррелятов депрессии у этих пациентов.

Результат: Из 265 обследованных пациентов (средний возраст 36,9±10 лет), 136 (51,3%) составляли женщины. Распространенность депрессии была 61.1% (95% CI: 55.1–66.8), со значительной долей (36,6%), имеющей умеренную депрессию. Многовариантный логистический регрессионный анализ показал, что принадлежность к женскому полу (a*OR*=3.0, 95% *CI* (1.7–5.5), *P*<0.001), с семейной историей психического заболевания (a*OR*=2.5, 95% *CI*: 1.3–5.4, *P*>0.05), повторный курс лечения ТБ (a*OR*=11.2, 95% *CI*: 5.2–31.1, *P*<0.001), досрочно завершенное лечение (a*OR*=8.2, 95% *CI*: 1.1–23.3, *P*<0.05) и ВИЧ/ТБ смешанная-инфекция (a*OR*=2.5, 95% *CI*: 1.2–6.5, *P*<0.001) является факторами, связанные с наличием больше шансов депрессии.

Заключение: Наше исследование показывает, что существует высокая распространенность депрессии среди больных ТЛ, более чем один из двух страдающих пациентов. Комплексный уход за больными туберкулезом с участием практикующих в области психического здоровья настоятельно рекомендуется, особенно для групп высокого риска.

Translated from English version into Russian by Turdimurot Rakhmonov, through

Prevalencia y correlaciones de los síntomas depresivos en pacientes adultos con tuberculosis pulmonar en la Región Sudoeste de Camerún

Jules Kehbila, Cyril Jabea Ekabe, Leopold Ndemnge Aminde, Jean Jacques N Noubiap, Peter Nde Fon, Gottlieb Lobe Monekosso

Resumen

Antecedentes: La tuberculosis (TB) continúa siendo un desafío de salud a nivel mundial y la depresión contribuye de manera significativa a la carga mundial de la enfermedad. La evidencia actual sugiere que existe una correlación entre los síntomas de depresión y la TB, la falta de cumplimiento con el tratamiento y un aumento en la morbilidad y mortalidad. Sin embargo, en Camerún no existe mucha información en cuanto a dichas correlaciones. El presente estudio tuvo como objetivo determinar la prevalencia y correlaciones de la depresión en pacientes adultos con TB pulmonar en la Región Sudoeste de Camerún.

Métodos: Entre el 2 de enero y el 31 de marzo de 2015 se llevó a cabo un estudio transversal hospitalario con 265 pacientes con TB pulmonar en el Hospital Regional Limbe y el Hospital del distrito Kumba. Se diagnosticó la depresión mediante el uso de un cuestionario de salud estándar de nueve puntos y se clasificó a la misma como inexistente, leve o moderada. Se utilizaron regresiones logísticas para investigar las correlaciones de la depresión en dichos pacientes.

Resultados: De los 265 pacientes (edad promedio 36,9±10 años) estudiados, 136 (51,3%) fueron mujeres. La prevalencia de la depresión fue del 61,1% (95% *IC*: 55,1–66,8) y una proporción significativa (36,6%) tenía depresión leve. Los análisis de regresión logística multivariante mostraron que el ser mujer (RM=3,0, 95% *IC* (1,7–5,5), *P*<0,001), tener antecedentes familiares de enfermedad mental (RM=2,5, 95% *IC*: 1,3–5,4, *P*>0,05), estar en tratamiento para la TB (RM=11,2, 95% *IC*: 5,2–31,1, *P*<0.001), haber discontinuado el tratamiento (RM=8,2, 95% *IC*: 1,1–23,3, *P*<0.05) y haber tenido una co-infección de VIH/TB (RM=2,5, 95% *IC*: 1,2–6,5, *P*<0.001) fueron factores que se asociaban a una mayor probabilidad de depresión.

Conclusión: Nuestro estudio sugiere que existe una prevalencia alta de depresión entre pacientes con TB pulmonar, con más de uno por cada dos pacientes afectados. Se fomenta ampliamente el cuidado multidisciplinario de pacientes con TB con la asistencia de especialistas en salud mental, en especial para los grupos de alto riesgo.

Translated from English version into Spanish by Maria Alejandra Aguada, through

TRANSLATORS
WITHOUT BORDERS
